# Modelling Discrete Choice Variables in Assessment of Teaching Staff Work Satisfaction

**DOI:** 10.1371/journal.pone.0115735

**Published:** 2015-04-07

**Authors:** Mihai Mieilă, Constanţa Popescu, Ana-Maria Tudorache, Valerică Toplicianu

**Affiliations:** Faculty of Economic Sciences, Valahia University, 35 Lt. Stancu Ion, Targoviste, 130105, Romania; University of Westminster, UNITED KINGDOM

## Abstract

Levels of self-reported job satisfaction and motivation were measured by survey in a sample of 286 teachers. Using the discrete choice framework, the paper tries to assess the relevance of the considered indicators (demographic, social, motivational) in overall teaching work satisfaction. The findings provide evidence that job satisfaction is correlated significantly with level of university degree held by the teacher, type of secondary school where the teacher is enrolled, revenues, and salary-tasks adequacy. This is important for the Romanian economy, since the education system is expected to provide future human resources with enhanced skills and abilities.

## Introduction

The educational system is a crucial link in transmitting the acquired knowledge between the past and future generations. It is largely considered that the current state of education influences dramatically the long-run (about 20 years) status of national community. Teaching staff motivation is one of the determinants of the educational system, affecting to some extent the behaviour of the actors involved in the educational process.

First, the teaching staff motivation is about motivating potential teachers. Due to the shortage of teaching staff, there are three aspects that concern the specialists: i) too few people entering the specialty studies for a teaching career; ii) too many beginner teachers who leave the educational system; and, iii) too many specialized graduates who does not want to practice. In the literature, there were identified three driving forces that influence behaviour of potential teachers: internal forces (or intrinsic personality related to teaching), external forces (environmental or extrinsic), and altruistic forces (internalized extrinsic) [1]. Based on these forces, the intrinsic motivation of potential teachers is influenced by: the reasons for which they prefer a teaching career; the probability of finding a job after graduation; the individual expectations and goals; and by the appetence of continuing their education through a master’s program [2]. Regarding the motivational aspects, other researchers consider that intrinsic motivation is prevalent in choosing a teaching career. Therefore, the prospective teachers having a stronger intrinsic motivation are expected to have a longer practice in the field and with better results [3]. Other authors try to list the factors that influence the motivation of prospective teachers. Among the most mentioned factors in this respect, are: the positive opinion regarding own teaching skills and abilities, pleasure of working with students and intellectual stimulation implied by the profession [4]; the subject expected to be taught, which would give the opportunity to capitalize the expertise [5]; or cultural background of aspirant teachers [6]. Studying the situation of specialized graduates who does not want to practice, some authors argue that certain experiences during their studies lead the young people to ignore the option for a career in education. In contradistinction to this approach, other papers study the motivation of people to leave their current job to become teachers [7].

From the point of view of the current situation in education, the most urgent problem is motivating teaching staff in service. There are extensively researches that appraise the applicability of motivational theories in the educational system [8–14]. Newer researchers argue that teachers are a category of employees of whose work motivation and performance are influenced by a multitude of motivating and demotivating factors. According to these studies, the factors involved in teaching staff motivation are established upon the principle of *thesis-and-antithesis*, and their importance varies among different persons [13, 15–21]. Other researchers turned their attention to the relationship between motivation, job satisfaction, and teachers’ performance. The results of these studies show that job satisfaction and motivation of teachers are significantly correlated with the level of responsibility, gender, subject taught, age, seniority, and the type of activity [22–24]. The analysis of the similarities and discrepancies between the teachers who work at different stages of education (primary, secondary and academia)—in terms of job satisfaction and motivation to remain in the education system—emphasize the increased importance granted by teachers to professional and interpersonal factors to the detriment of practical ones [25]. There are some researches that examine the correlation between developments in salaries of university graduates and teachers. A model developed in this respect outlines that, as salaries of graduates increase (phenomenon known as "rising talent premium"), the competent authorities shall adjust the salaries of teachers and quality standards [26]. However, often this does not occur, which determines many graduates and teachers in service to opt for the business sector, which offers better financial rewards. At the same time, the analysis of performance-based payment system in education shows that this type of payment has led to improvements in the results of schools, students, and teachers [27]. Nevertheless, in trying to solve these problems, teachers’ motivation should not be affected, realizing that the way the teachers are treated today determines the future of the educational system.

Important lessons can be learned form researches focused on motivation factors that lead teachers to leave the educational system [19]. Thus, the most mentioned reason of resignations is the unrealistic expectations of future teachers—as their expectations do not match the actual teaching activity. As other papers point out, the human resources policies in education have to be accompanied by the full transparency and advice of future teachers on the educational system realities, in order to develop personal goals consistent with the specific of profession and outline realistic expectations [28].

## Materials and Methods

### Ethic statement

The research was approved by the scientific board of the Faculty of Economics—Valahia University.

### Study methodology

The underlying idea of the model is that motivation is one of the main factors of influence upon job satisfaction. Consider a teacher *i* and, taking into account the literature above presented, assume the following motivation factors that determines work satisfaction (*WS*) reported by the interviewee: *AGE* = teacher age (in years); *DEG* = the professional degree the teacher holds; *EDU* = education (university) degree of the teacher; *LYC* = type of secondary school where respondent teacher works; *SRTY* = total work history (in years); *TEACH* = amount of experience in education (in years); *SAL* = satisfaction reported by the interviewee from the salary point of view; *FAIR* = satisfaction reported by the teacher about the salary adequacy with regard to efforts for tasks’ accomplishment; *PREM* = satisfaction reported by the interviewee regarding the revenues received as premium.

The importance of demographic factors in work satisfaction is widely emphasized in literature [22–24, 29–30]. As a consequence, in the present paper, two measures for age (age and total work history) and two measures for amount of experience in education (teaching length of service and professional degree) will be used. The two measures for age are correlated, since seniority is expected to increase with the age; in our study, this statement is confirmed by the computations results. Analogue, for teachers enrolled in Romanian education system, is very usual to hold a teaching degree consistent with teaching length of service; also, in case of present study, the field situation is consistent with this assertion. Thus, these measures have to be used alternatively, giving the following forms of the model:

$$WS_{1}=f\left(DEG,EDU,LYC,SRTY,SAL,FAIR,PREM\right)$$ (1)

$$WS_{2}=f\left(AGE,EDU,LYC,TEACH,SAL,FAIR,PREM\right)$$ (2)

In the Eq 1 and Eq 2, the endogenous variable, besides some exogenous variables, are limited dependent variables. The reasons for which the econometric modelling of this type of variables through the OLS regression model is not suitable are largely detailed in the literature [31–34]. The estimation of the model is set on the residual variable distribution function. Therefore, if is considered that the endogenous variable follows the normal distribution, *probit* methods may be employed; considering that the variable is logistic distributed, the *logit* model is the choice; in the same time, the *Weibull* distribution may be useful as well. The *probit* model is based on the normal cumulative distribution function, $\Phi \left(z\right)=\int_{-\infty }^{z}\varphi \left(x\right)dx$, where $\varphi \left(z\right)=e^{-z^{2}/2}/\sqrt{2\pi }$, whereas the *logit* model is based on the logistic cumulative distribution function Λ(*z*) = 1/(1 + *e* ^−*z*^) = *e* ^*z*^/(1 + *e* ^*z*^).

Specifically, in the case of multinomial ordered choices, the values of endogenous variable are subject of modelling through considering a latent (unobserved) variable, $y_{i}^{*}=\beta^{\prime }x_{i}+\varepsilon_{i}$, $i=\bar{1,n}$, *ε*_*i*_ ∼ *N*(0, 1), *β* is a vector of parameters, not containing an intercept. The introduction of the latent variable within the model relies on the impossibility of its direct observation. In such cases, an individual chooses a certain level of satisfaction if the difference of utility exceeds a certain threshold level, depending on the overall exogenous variables, $X_{p},p=\bar{1,k}$ [35].

$$y_{i}=\left\{ \begin{array}{l} \;0,\text{ if }y_{i}^{*}\leq \delta_{0}\\ \;1,\text{ if }\delta_{0}<y_{i}^{*}\leq \delta_{1}\\ \;2,\text{ if }\delta_{1}<y_{i}^{*}\leq \delta_{2}\\ \;J,\text{ if }\delta_{J-1}\leq y_{i}^{*} \end{array}\right.$$ (3)

The *δ*-s represent the unknown "threshold"-parameters that are estimated along with *β* coefficients, by maximizing the log-likelihood function, which requires that *ε*-s are assumed to be distributed either as a standard normal (in case of ordered *probit*), either the cumulative density of *ε* is the logistic function (and the modelling is done through the ordered *logit*).

Results the probability for the unit *i* of the population to belong to one of the considered classes:

$$\text{Pr}\left(y_{i}=j|x_{i}\right)=\text{Pr}\left(\varepsilon_{i}\leq \delta_{j}-\beta^{\prime }x_{i}\right)-\text{Pr}\left(\varepsilon_{i}<\delta_{j-1}-\beta 'x_{i}\right)=\Phi \left(\delta_{j}-\beta 'x_{i}\right)-\Phi \left(\delta_{j-1}-\beta 'x_{i}\right),j=\bar{1,J}$$ (4)

where Φ(*z*) represents the cumulative standard normal density function, Pr(*A*) is the probability of the event *A*, $\sum_{i=1}^{J}\text{Pr }i=1$, and (for all the probabilities to be positive) 0 < *δ*_1_ < *δ*_2_ <…< *δ*_*J*−1_.

In this study, the estimation of the model parameters and of the variance matrix is based on second analytic derivative of the above function, using the optimizing algorithm Goldfeld-Quandt [36]. Specific to this method is that the convergence of a maximum is achieved in the absence of *a priori* knowledge regarding the strictly concavity of the function.

Usually, the marginal effects of the regressors **x** on the probabilities are not equal to the coefficients. For the probabilities above presented, the marginal effects of changes in the regressors may be described as

$$\frac{\partial \text{Pr}\left(y=j{|x}_{i}\right)}{\partial x_{i}}=\left[\phi \left(\delta_{j-1}-\beta^{\prime }x_{i}^{\prime }\right)-\phi \left(\delta_{j}-\beta^{\prime }x_{i}\right)\right]\beta ,\text{ with }\;\sum \frac{\partial \text{Pr}\left(y=i|x\right)}{\partial x}=0$$ (5)

The validity of the *probit* estimator can be tested after estimation. However, some issues arise when using conventional *R* ^2^-type measures. Specific to the discrete choice models is the existence of a variety of measures for goodness-of-fit, extensively described in the literature [31–32, 37]. In this study, in appraisal of the estimations accuracy, were employed two measures: *pseudo*−*R* ^2^ and the likelihood ratio test.

$$pseudo\text{-}R^{2}=1-\frac{N}{N+2\left(\text{ln }L_{U}-\text{ln }L_{R}\right)}$$ (6)

where N represents the number of observations. As *L*_*R*_ < *L*_*U*_, the larger difference between the two likelihood values, the more extended ads to the very restrictive model.

The likelihood ratio test is a common test, similar to the *F* test that all the slopes in a regression are zero:

$$LR=-2\left[\text{ln }L_{R}-\text{ln }L_{U}\right]$$ (7)

where *L*_*U*_ denotes the maximum (unrestricted) likelihood value of the model of interest, and *L*_*R*_ represents the maximum value of the likelihood function when all parameters, except the intercept are set (restricted) to zero.

### Study setting and sampling

The data set used in this study was collected from a special organized research in secondary schools of Dambovita County. Specific to the Romanian educational system is the division of high schools in categories upon their main specific. According to this classification, at our study took part teachers from five national secondary schools, five theoretic high-schools, and five technologic secondary schools. Due to limited resources allocated to the research and because of unavailability of an accurate survey basis for building the sample, we used the multistage survey. In the first stage were extracted randomly, by the above mentioned types, the sample of secondary schools of all high schools in the area under investigation. In the next stage, the teachers of each school who responded to the questionnaire were chosen using the step counting method.

According to the Report regarding the state of school education in Dambovita County, for the first semester of the school year 2012–2013, were enrolled 1358 high school teachers. Based on this information, the initial research sample was set to 300 people, in order to ensure a significance level of 5%. However, in order to be protected against a too high rate of non-response, which could jeopardize the viability of the research, and to keep a uniform unit of observation, we determined the appropriate research sample of 375 teachers (25 teachers from each school under review).

Of the 375 teachers approached, 331 returned back completed questionnaires, i.e. an 11.7% ratio of non-responses. As 55 questionnaires indicate “do not know / no answer” for the dependant variable, that is, the work satisfaction, they could not be used in the model, as there was not possible to assign a specific value for this situation. Therefore, 276 questionnaires were used in the modelling process framework, as described above. Table 1 contains the main features of the considered sample in comparison with the statistic data for the teachers working in secondary education of Dambovita County.

**Table 1:** The main features of the considered sample vs. the actual data.

	Sample	Actual
***Age of teachers***		
**Mean**	41	43
**Standard Deviation**	9.4	11.7
***Teaching experience (in years)***		
**Mean**	16	17
**Standard Deviation**	9.35	11.9
***Teaching degree***		
**Debutant**	0.0664	0.0725
**Definitive**	0.1853	0.1947
**Second degree**	0.2168	0.2314
**First degree**	0.5315	0.5014
***Education degree***		
**University**	0.4582	0.4306
**Post-university/M.sc.**	0.4774	0.4992
**PhD**	0.064	0.0702
***Categories of subjects taught***		
**Romanian**	0.1434	0.152
**Modern Languages**	0.1503	0.1324
**Mathematics**	0.1154	0.1244
**Computer Science**	0.0804	0.0864
**Humanities**	0.2622	0.2438
**Sciences**	0.1574	0.1631
**Technologies**	0.0909	0.0979

Table 1 shows the comparison between the main statistical features of the research sample and of the data regarding the whole teachers population enrolled in the high schools in Dambovita County.

As the deviation between the averages of the two series of data (actual and sample) does not exceed 10%, and taking into account that in the survey the age and the experience were not provided exactly, but upon certain ranges (Table 2), this may be considered an accurate representation of studied population. Table 2 presents the summary of collected data and values assigned in model.

**Table 2:** Summary of general information and values assigned in the model^a^.

			Value assigned in the model					Teachers (N = 276)		
***Work satisfaction***										
**Very unsatisfactory**			1					0.0145		
**Rather unsatisfactory**			2					0.1486		
**Rather satisfactory**			3					0.6739		
**Very satisfactory**			4					0.1630		
***Age of participant teachers***										
**Under 30**			25					0.0909		
**30–40**			35					0.4231		
**41–50**			45					0.2517		
**Over 50**			55					0.2343		
***Teaching degree***										
**Debutant**			1					0.0664		
**Definitive**			2					0.1853		
**Second degree**			3					0.2168		
**First degree**			4					0.5315		
***Education degree***										
**University**			1					0.4582		
**Post-university/M.sc.**			2					0.4774		
**PhD/ Post-doctoral**			3					0.064		
***Type of Secondary School***										
**Technologic Sec. School**			1					0.3442		
**Theoretic Sec. School**			2					0.2862		
**National Sec. School**			3					0.3696		
*Lenght of service*									Teachers (N = 276)	
***Years***	Value assigned in the model			*Total working history*			*Years of teaching experience*			
**0–5**	2.5			0.0804			0.0979			
**5–10**	7.5			0.1364			0.1888			
**10–15**	12.5			0.2448			0.2448			
**15–20**	17.5			0.1538			0.1469			
**20–25**	22.5			0.1084			0.0979			
**25–30**	27.5			0.0979			0.0804			
**30–35**	32.5			0.1783			0.1434			
*Salary satisfaction reported*						Teachers (N = 276)				
		Value assigned in the model			*Salary*	*Tasks-Salary Adequacy*				*Premiums*
**Total disagreement**		1			0.2810	0.2847				0.5328
**Disagreement**		2			0.1715	0.2190				0.1314
**Rather disagreement**		3			0.2445	0.2263				0.0803
**Neutral**		4			0.1788	0.1533				0.0876
**Rather agreement**		5			0.1095	0.0985				0.0620
**Agreement**		6			0.0146	0.0182				0.0693
**Total agreement**		7			0.2810	0.2847				0.5328

Table 2 shows the summary of general information regarding the weight of provided answers by items, and values assigned in the model.

^a^ The full dataset is available from the authors on request.

The questionnaire used in the survey was structured as follows: (i) First section comprise 40 pre-coded items on professional, financial, and other sources of motivation. In assessing the answers to these items, there was used Likert’s scale. This section covers the main part of the questionnaire. (ii) Second section consist of three (two open and one closed) questions, which aims to identify different motivational experiences and knowledge of the general level of job satisfaction of interviewed teachers. (iii) The last section questionnaire is dedicated to the items regarding social, demographic, professional, and contextual aspects of the teachers participating in the survey. These information are used in statistical processing of the questionnaire. Table 3 displays the descriptive statistics of the variables.

**Table 3:** Descriptive statistics.

	*WS*	AGE	DEG	EDU	LYC	SRTY	TEACH	SAL	FAIR	PREM
**Mean**	2.9855	41.2319	3.1957	2.0181	2.0254	17.7899	16.2138	2.7391	2.6486	2.4022
**Median**	3	35	4	2	2	17.5	12.5	3	2.5	1
**Max.**	4	55	4	4	3	32.5	32.5	7	7	7
**Min.**	1	25	1	1	1	2.5	2.5	1	1	1
**Std. Dev.**	0.6089	9.3965	0.9713	1.0285	0.8460	9.4536	9.3553	1.4438	1.4333	1.8842
**Skewness**	-0.3804	0.1284	-0.8283	0.2651	-0.0480	0.1946	0.4064	0.3819	0.5501	1.1147
**Kurtosis**	3.9441	1.9927	2.4155	1.5155	1.4039	1.9101	2.0777	2.2998	2.5091	2.9084
**Jarque-Bera**	16.906	12.428	35.489	28.577	29.403	15.401	17.378	12.349	16.692	57.257
**Prob.**	0.0002	0.0020	0.0000	0.0000	0.0000	0.0005	0.0002	0.0021	0.0002	0.0000
**Sum**	824	11380	882	557	559	4910	4475	756	731	663
**Sum Sq. Dev.**	101.94	24281.16	259.435	290.91	196.82	24576.81	24068.39	573.22	564.91	976.36
**Obs.**	276	276	276	276	276	276	276	276	276	276

Table 3 presents descriptive statistics of the considered variables.

## Results and Discussion

In order to determine the working satisfaction of the teachers participant at the survey, were considered the following demographic variables: teaching degree (*DEG*) and total length of service (*SRTY*) in Eq 1, respectively age (*AGE*) and amount of experience in education (*TEACH*) in Eq 2. This alternative use of two proxies for the same demographic variable is designed to ensure a double-check correlation between the work satisfaction and the two considered variables (age and seniority). The estimations use ordered *probit* and *logit* techniques and they are performed through using of Eviews software package. Tables 4 and 5 contain the reported results.

**Table 4:** Estimated Work Satisfaction: Eq 1 (DEG and SRTY).

Variables	*PROBIT* Coefficient	*LOGIT* Coefficient
**DEG**	− 0.0197 (− 0.218)	− 0.0223 (− 0.136)
**EDU**	0.1521** (2.101)	0.2749** (2.087)
**LYC**	0.2684** (3.03)	0.44*** (2.742)
**SRTY**	0.0008 (0.082)	0.0005 (0.03)
**SAL**	0.1695** (2.252)	0.3213** (2.4)
**FAIR**	0.1475** (1.979)	0.2808** (2.154)
**PREM**	0.0327 (0.775)	0.0394 (0.507)
**Pseudo *R*** ^**2**^	0.1025	0.103
***LR* statistic**	51.293^(^ ***	51.485***
**Log-likelihood**	− 224.495	− 224.398
**Restr. log likelihood**	− 250.140	− 250.140
**Avg. log likelihood**	− 0.813	− 0.813
**−*β’x***_***i***_	− 0.7212* (− 1.862)	−1.5454** (−1.984)
**Δ**_**1**_ ***−β’x***_***i***_	0.6101* (1.717)	1.1757* (1.856)
**Δ**_**2**_ ***−β’x***_***i***_	2.8549*** (7.244)	5.0491*** (6.86)

Table 4 displays the values of the estimated variables taking into account the professional degree hold by the teacher (= *DEG*) and total work history (in years) (= *SRTY*) as proxies for the demographic factors.

Note: the values in brackets are the *z* statistics.

Significance at the 1, 5, and 10% levels is denoted by ***, **, and *, respectively.

**Table 5:** Estimated Work Satisfaction: Eq 2 (AGE and TEACHING).

Variables	*PROBIT* Coefficient	*LOGIT* Coefficient
**AGE**	− 0.0012 (− 0.09)	− 0.0626 (− 0.255)
**EDU**	0.1493** (2.061)	0.2711** (2.054)
**LYC**	0.266** (3.01)	0.4347*** (2.707)
**TEACHING**	− 0.0012 (− 0.089)	0.0028 (0.113)
**SAL**	0.1694** (2.254)	0.3194** (2.384)
**FAIR**	0.1477** (1.983)	0.2814** (2.160)
**PREM**	0.0319 (0.758)	0.0388 (0.500)
**Pseudo *R*** ^**2**^	0.103	0.103
***LR* statistic**	51.332***	51.557***
**Log-likelihood**	− 224.474	− 224.361
**Restr. log likelihood**	− 250.140	− 250.140
**Avg. log likelihood**	− 0.813	− 0.813
**−*β’x***_***i***_	− 0.7554 (− 1.399)	− 1.723* (− 1.671)
**δ**_**1**_ ***−β’x***_***i***_	0.577 (1.123)	1.0001 (1.091)
**δ**_**2**_ ***−β’x***_***i***_	2.823*** (5.224)	4.875*** (4.956)

Table 5 displays the values of the estimated variables considering the age of the teacher (= *AGE*) and the amount of experience in education (= *TEACH*) (both in years) as proxies for the demographic factors.

Note: the values in brackets are the *z* statistics.

Significance at the 1, 5, and 10% levels is denoted by ***, **, and *, respectively.

The output results using the *probit* and *logit* estimations are very similar, proofing the assertion that, generally these two forms give similar predictions [31]. Variables that reported statistically significant influence (at 5% level of significance) upon teachers work satisfaction are: *LYC* (secondary school type) – 1% according to *logit* model; *EDU* (university degree of the teacher); *SAL* (satisfaction reported by the interviewee from the salary point of view); and *FAIR* (satisfaction reported by the teacher about the salary adequacy with regard to efforts for tasks’ accomplishment. Despite the importance granted to demographic variables and special design of the study in order to ensure a proper measure of their influence, the reported values are not statistically significant. This may be considered as inconsistent with some of above mentioned studies, eg. [22–24]. According to other points of view expressed in the literature, the job satisfaction starts at high level, decline, and then starts to improve with increasing of the age, following a *U*-shaped curve [8]. Therefore, as the age or seniority on the one hand, and the job satisfaction, on the other hand, are variables that do not evolve in a similar or inverse manner, there may not be established a relationship neither positive nor negative between them.

Specific to the discrete choice models is that neither the sign nor the value of the parameters is informative about the estimation results, thus, direct interpretation of the parameters is ambiguous. The coefficients of the variables reported as significant through the model are subject to future processing, in order to establish their true sense, that is, the marginal effects. Using the Eq 5 the coefficients are evaluated using standard normal densities at the „threshold”-points φ(*δ*_*j*_). The resulting coefficients from Eq 1 using *probit* model (Table 4) are subject of this processing (as all the „threshold”-points *δ*_*j*_ reported statistically significant values). The estimates for the *probit* model imply

$$\begin{array}{l} y*=-0.0197\;Deg+0.1521\;Edu+0.2684\;Lyc+0.0008\;Srty+0.1695\;Sal+0.1475\;Fair+0.0327prem\\ y*=\left\{ \begin{array}{l} 0,\;if\;y*=0\\ 1,\;if\text{ 0}<y*\leq 1.3313\\ 2,\;if\;1.3313<y*\leq 3.571\\ 3,\;if\;y*>3.571 \end{array}\right. \end{array}$$

The results are presented in the Table 6.

**Table 6:** Estimation of conditional probabilities and marginal effects.

	Φ	Marginal effects			
		EDU	LYC	SAL	FAIR
y* = 0	0.2354	− 0.0468	− 0.0826	− 0.0399	− 0.0347
y* = 1	0.7291	− 0.0036	− 0.0063	− 0.0837	− 0.0381
y* = 2	0.9978	0.0494	0.0871	− 0.0456	0.0332
y* = 3	1.0000	0.0010	0.0018	0.1691	0.0397

In the Table 6 are presented the estimated values of conditional probabilities and marginal effects for the variables that reported significant influence.

**Fig 1:**
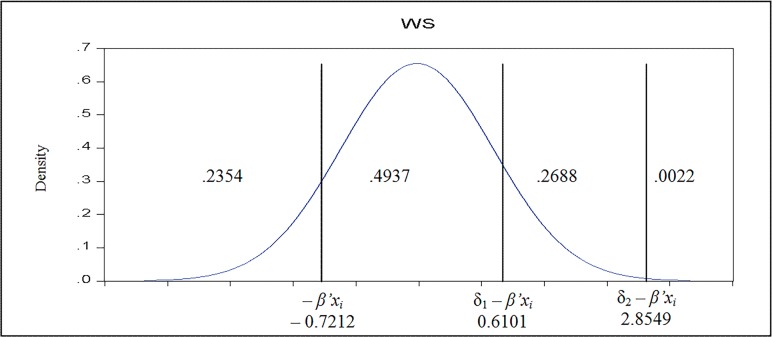
Estimated Ordered *Probit* Model. The plot depicts the work satisfaction implied model for teacher with average characteristics (by taking into account the considered variables).

The marginal effects express the influence upon the specific probabilities per unit change in the regressor; it depends on all the parameters considered in the model, the data, and which probability (cell) is of interest. It can be negative or positive. The figures in the Table 6 show the implied model for a teacher with the following characteristics: university degree of 2.018 (~ M.sc. degree), a professional degree of 3.196 (~ second grade), type of high school = 2.025 (~ theoretical), a total work history of 17.75 years, reporting average salary satisfaction (2.739), a tasks-salary adequacy (2.649) and financial incentives received (2.402). At the change in characteristics (**x**), the probability distribution changes accordingly. In terms of the figure, changes in the characteristics induce changes in the placement of the partitions in the distribution and, in turn, in the probabilities of the outcomes [37]. Fig 1 displays the work satisfaction implied model for a person with average characteristics, as described above.

**Fig 2:**
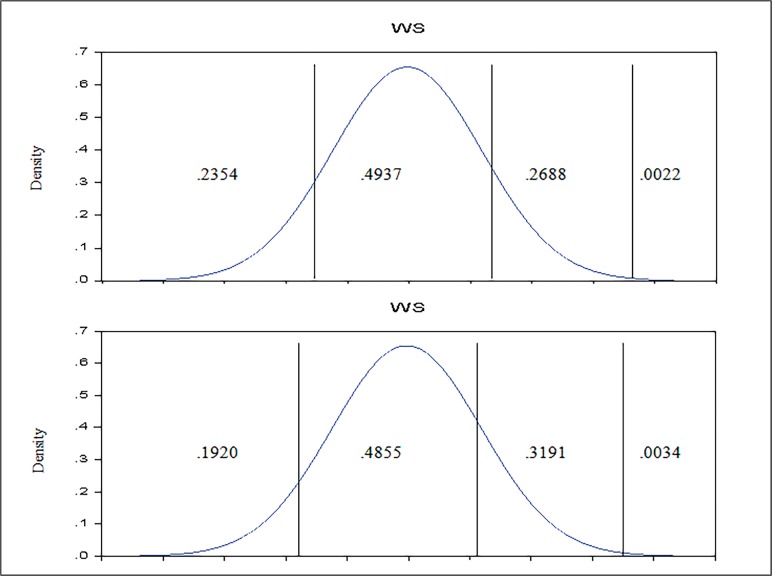
Partial effect of increase of educational degree (to PhD) in Ordered *Probit* Model estimated. The plot below depicts the work satisfaction implied model for a teacher who holds a PhD degree (all the other characteristics maintained unchanged, that is, at average level), in comparison with a teacher with average characteristics (in the plot above).

As appears in the Table 6, the partial effects of expected change on the probabilities per additional degree of education are: -0.0468, -0.0036, 0.0494, and 0.0010, respectively. Roughly speaking, an improvement in university degree status to PhD, is more likely to lead to improvements in work satisfaction; since the effect are denominated as “marginal”, the changes implied are typically of reduced magnitude, as it appears in the Fig 2.

**Fig 3:**
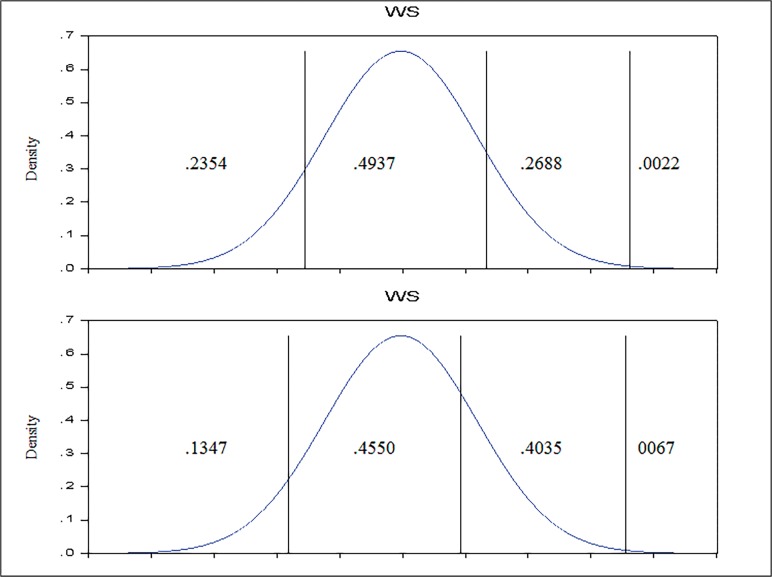
Partial effect of increase (to 5) of salary satisfaction reported in Ordered *Probit* Model estimated. The plot below depicts the work satisfaction implied model for a teacher reporting the salary as *rather satisfactory* (all the other characteristics maintained unchanged, that is, at average level), by comparison with a teacher with average characteristics (in the plot above).

According to the figures displayed in the Table 6, the situation shown in Fig 2 is common for the other significant variables, except the revenues. Regarding the salary satisfaction reported, it is interesting to remark that though the salary satisfaction reported is above mean, it has a negative effect over the work satisfaction; only for the teachers reported their job as very satisfactory, every new step in salary contempt is expected to lead to an increase in work satisfaction by 16.91%. If we consider the same individual shown in Fig 1, except now, with a salary satisfaction reported of five, the probability distribution for job satisfaction is presented in the Fig 3.

This situation may be also the result of a dissimulated reporting of job satisfaction concomitant with reduced levels of salary contempt; the statistical significance of revenues satisfaction within the model of work satisfaction expresses that type of reporting is sizeable.

## Conclusions

The quality of education significantly influences the general situation of national community in long-run and may be also considered as an important economic issue. The future status of Romanian emergent economy, besides its’ societal facet, is mostly dependent on the quality of graduates as the main channel providing human resources for companies. Improvements in motivation of teaching staff may lead to enhanced skills and abilities of future human resources. In assessment of teacher work satisfaction, the present paper uses ordered choice approach and employs the estimation method exposed in [36]. To the authors’ best knowledge, this is the first study that examines the work satisfaction of teaching staff in Romania using discrete choice approach. The findings in present study may contribute to relevant literature as evidence for work satisfaction in education and may serve as a model for sectoral researches.

**Fig 4:**
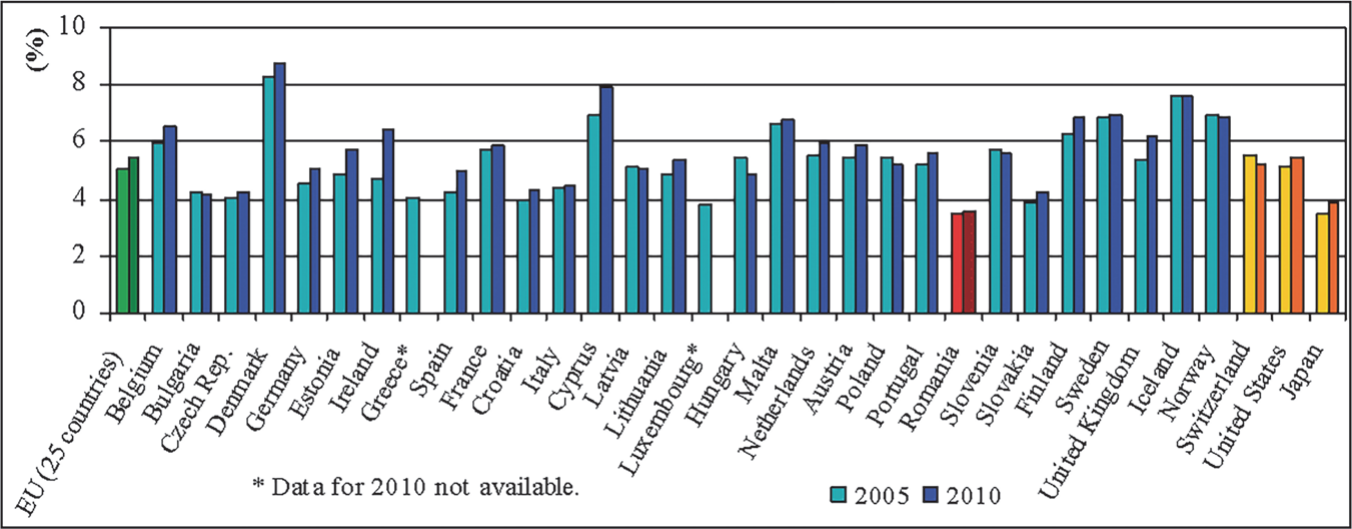
Total public expenditure on education in European Economic Area countries, United States and Japan as % of GDP, for all levels of education combined. Romania has the lowest allocation as % of GDP for education from all the considered countries. *Data source*: Eurostat.

**Fig 5:**
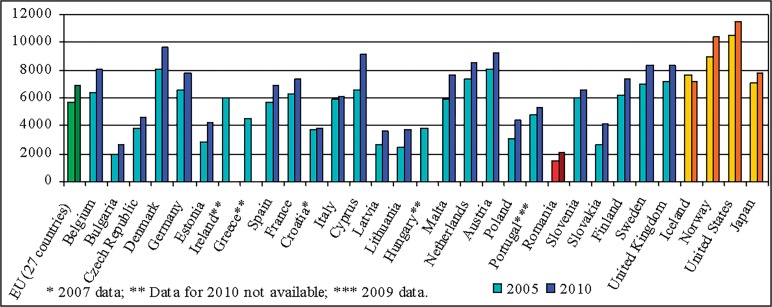
Annual expenditure on public and private educational institutions per pupil/student, 2005 and 2010 (by level of education—PPS based on full-time equivalents). Romania has the lowest allocation for education per pupil/student in all the considered countries. *Data source*: Eurostat.

**Fig 6:**
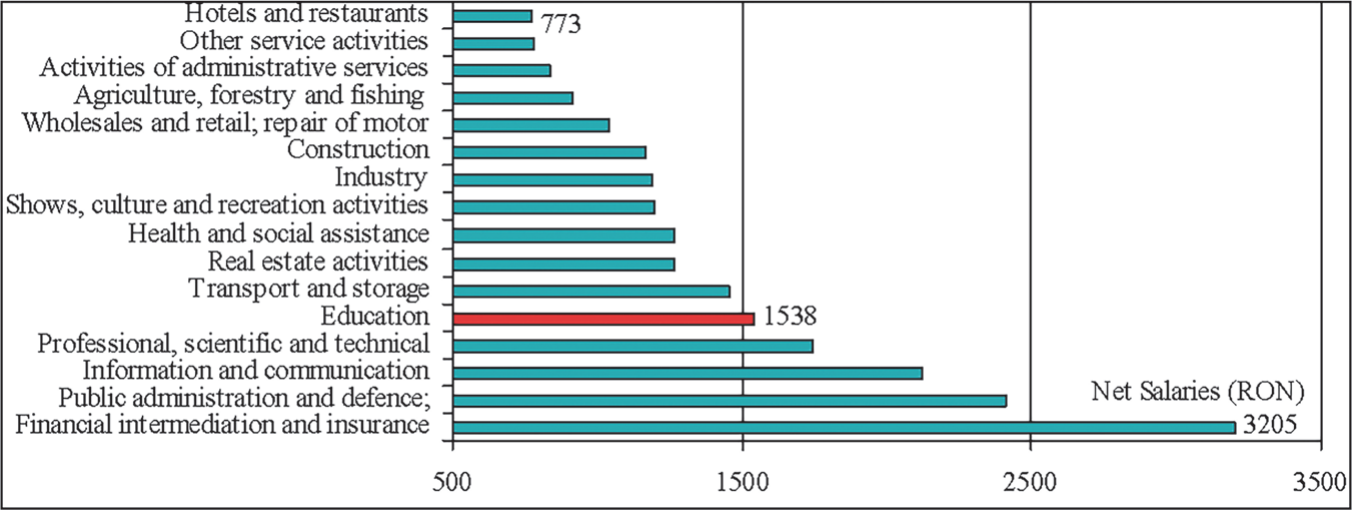
Hierarchy of average net nominal monthly earnings in Romania in 2008, by CANE section. The education occupies the fifth rank in this hierarchy. Average exchange rate in 2008: 1 Euro = 3.6826 RON (European Central Bank). *Data source*: National Institute of Statistics.

**Fig 7:**
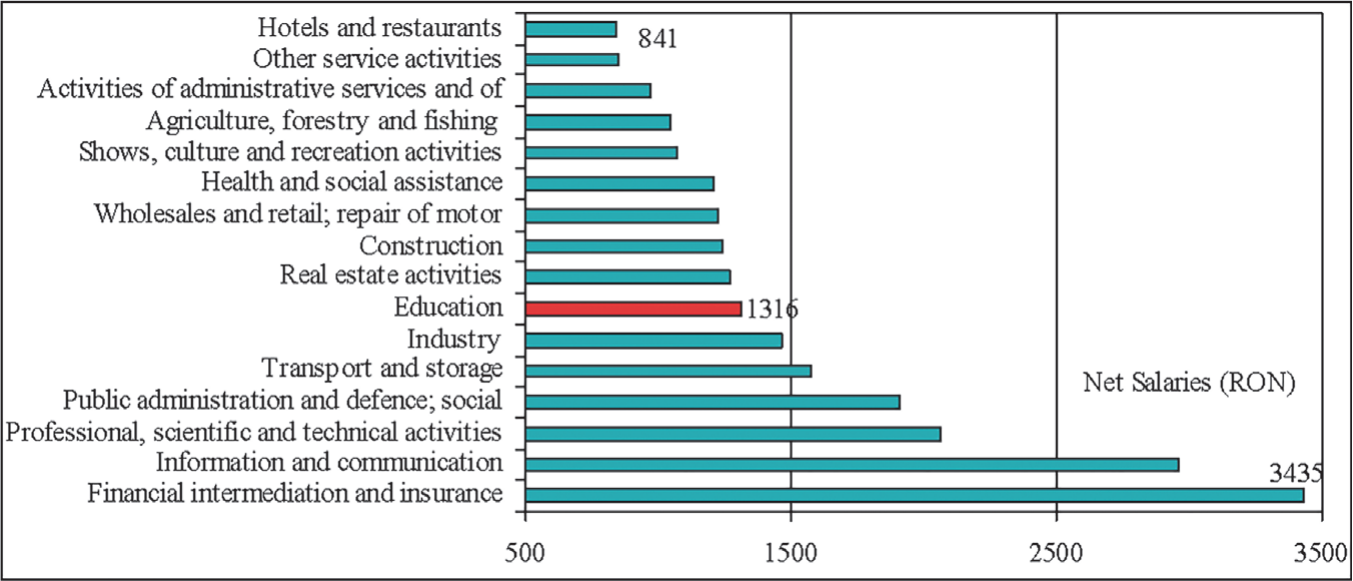
Hierarchy of average net nominal monthly earnings in Romania in 2011, by CANE section. The education occupies the seventh rank in this hierarchy, concomitant with a reduction in average net nominal monthly salaries of 14.34% (26.6% in Euros). Average exchange rate 2012: 1 Euro = 4.2391 RON (European Central Bank). *Data source*: National Institute of Statistics.

The results indicate the salary as an issue point in Romanian teaching staff motivation. This situation is the joint-result of reduced subsidies and incentives for sponsorship, as is depicted in the Figs 4 and 5, as well as of the place and evolution of salaries in education in general hierarchy of revenues (Figs 6 and 7). On the average, teachers report job as rather satisfactory though the salary influences negatively this satisfaction. This situation is consistent with the theory according to that job satisfaction results when intrinsic aspects of work (motivators; e.g. recognition, promotions, etc.) promote feelings of happiness in the worker, and job dissatisfaction results when the extrinsic factors (hygienes; e.g. salary, working conditions, etc.) are considered [8]. Though, this approach is criticised by findings of further studies that indicate that the same factors can cause both satisfaction and dissatisfaction [23, 38].

Overall, the results of the present study indicate a significant effect of the secondary school type, university degree, salary and adequacy between tasks and salary in teaching staff work satisfaction. Following previous researches that reported age and seniority as significant factors influencing personal work satisfaction of teachers, two different proxies for age and two proxies for years of teaching experience are used. Despite the special design of the study for adequate control the social and demographic variables, these factors do not seem to have an important influence upon job satisfaction. Since we found this evidence, the role of motivation is expected to become more critical within efforts for improvement the quality of education. However, the overall teaching staff (for all levels of education) has the largest share in the total state staff of Romania. To overcome the adverse financial and state budget developments due to unexpectedly and significant growth in teaching staff costs, the incentive instruments and private sponsorships should be encouraged by the authorities. In this way, improvements in quality education and economic recovery may be achieved in a way less dependent on the state budget. The results of this study may hopefully present an idea on the teaching staff motivation in similar economies. A future study covering a cross-country analysis may provide a broader idea on the teaching staff motivation factors in developing countries.
